# National Tissue Freezing Protocol is Essential for Modern Cancer Care and Research

**DOI:** 10.15761/tim.1000293

**Published:** 2026-04-02

**Authors:** Margaret Simonian

**Affiliations:** David Geffen School of Medicine, University of California Los Angeles

## Abstract

Modern cancer care and precision medicine increasingly rely on high quality human tissue yet tissue preservation practices in many healthcare systems remain outdated. Traditional formalin-fixed paraffin-embedded (FFPE) tissues while long established, is poorly suited to modern molecular and multi-omics technologies due to chemical degradation of DNA, RNA, and proteins. In contrast tissue freezing preserves molecular integrity and enables advanced proteomics, transcriptomics, genomics, and metabolomics analyses that are essential for accurate diagnosis, treatment selection, and biomedical research.

To address these disparities, there is a compelling case for adopting a fixed nationally agreed tissue freezing protocol such a protocol should be implemented across all hospitals and aligned with consent, storage, and transport standards. Without a standardized protocol valuable tissue samples may become unsuitable for modern diagnostics ultimately constraining both patient care and scientific advancement.

## Why a national tissue freezing protocol is essential for modern cancer care and research

Advances in precision medicine increasingly depend on access to high quality human tissue. Yet in many healthcare systems, tissue preservation practices have not kept pace with scientific progress. Tissue freezing (snap freezing, also known as flash freezing), offers a powerful alternative to traditional chemical fixation methods with formalin and is now essential for delivering proper cancer diagnosis, treatment, and research.

With tissue freezing, samples preserved by rapidly lowering their temperature anywhere from −80° C to −210° C in an Ultra-Low freezers or Liquid Nitrogen, preserving the molecular structural integrity of the tissue. Unlike formalin-fixed tissues (FFPE) which remains the current standard of care in many hospitals, frozen tissues are compatible with a wide range of modern molecular technologies. These include genomics, transcriptomics, proteomics, metabolomics, and other multi-omics approaches, which are increasingly central to precision medicine. Formalin fixation chemically alters proteins and nucleic acids, making it unsuitable or severely limiting for many emerging diagnostic and research techniques. As medicine moves toward data driven, molecularly guided care, reliance heavily on formalin alone risks leaving patients and researchers behind.

## Evidence supporting fresh-frozen (FF) Tissue over FFPE tissue samples

A growing number of research revealed that FF tissues and biopsies outperform FFPE samples in molecular and omics analyses. Multiple studies have demonstrated many advantages of FF over FFPE, in particular: the proteins/DNA/ RNA extracted from FFPE tissues are frequently highly degraded compared to FF samples; FF tissues exhibit less variability than FFPE tissues, as fixation methods can affect DNA and RNA preservation and overall data quality; the processes of fixation/embedding may result in deamination that damages DNA/RNA, leading to artifactual mutations or false negative results; FF samples can be stored for more than 2 years with no risk of DNA/protein degradation unlike FFPE samples. Additionally, some studies report that FFPE samples can show transcriptomic similarities and a high correlation of gene expression with frozen samples once the most degraded samples are excluded, this highlights a major limitation [1–5].

In real world clinical practice, fixation and embedding processes are not tightly controlled. Variables such as tissue thickness, fixative volume, and fixation duration (often ranging from 24 - 72 hours) vary substantially across institutions. These inconsistencies directly affect DNA and RNA integrity, increasing the risk of inaccurate or incomplete molecular results. Moreover, FFPE protocols are not standardized nationally or internationally. Samples collected at different times or in different hospitals may be processed in fundamentally different ways, introducing technical variability that undermines reproducibility and data comparability. This variability is particularly problematic for molecular genetic analysis, multi-omics research, and inter-hospital tissue sharing. In contrast, fresh-frozen tissue provides consistency, reproducibility, and molecular fidelity, making it the preferred substrate for precision medicine, translational research, and future proof diagnostics.

## The case for a fixed nationally standardized protocol for tissue freezing

To address these disparities, there is a compelling case for adopting a fixed, nationally agreed tissue freezing protocol, such a protocol should be implemented across all hospitals and aligned with consent, storage, and transport standards. This standardization would ensure equitable patient access to tissue freezing regardless of treatment location, enable safe and efficient sharing of frozen tissue between hospitals and research facilities, improve turnaround times for molecular testing, strengthen national and international research collaboration, and future-proof tissue samples for emerging omics technologies. Without a unified protocol, valuable tissue samples risk being unusable for modern diagnostics, limiting both patient care and scientific discovery.

Recently, the UK Parliament called for a debate on the standardization of tissue-freezing protocols, specifically for brain cancer, recognizing the urgent need to ensure equitable access to high-quality tissue preservation regardless of where patients receive care. This parliamentary focus reflects a growing consensus that fragmented and inconsistent tissue preservation practices are directly limiting patient access to advanced diagnostics, biomarker-driven care, and precision medicine. The UK’s approach demonstrates the critical value of nationally coordinated standards that guarantee quality, consistency, and interoperability across hospitals and research institutes. Other research leading nations, including the United States and Australia, should urgently implement mandated, standardized tissue-freezing protocols. Such policies would enable safe inter-institutional tissue sharing, reduce systemic inequities, and ensure that all patients can equally benefit from modern molecular and genomic technologies.

Having worked with numerous frozen and FFPE tissue specimens, I have consistently advocated for the collection and preservation of fresh-frozen tissues whenever feasible. Accordingly, I recommend and proposed that whenever tissue is obtained, whether during surgical resection or biopsy, an adequate quantity be collected to allow a portion to be preserved as fresh-frozen material. Such preservation enables subsequent applications in genetic and molecular testing, diagnostic evaluation, research studies, or treatment planning as new testing methods or therapeutic options evolve. Therefore, I strongly advocate for the adoption of a nationally standardized protocol for tissue freezing for all cancer types.

## Looking ahead

Tissue freezing is no longer a niche research tool, it is a cornerstone of modern precision medicine. As the limitations of formalin fixation become increasingly clear, healthcare systems must evolve. By establishing a regulatory framework and enforcing a standardized national tissue-freezing protocol, hospitals and research institutes can ensure that every patient’s tissue is preserved to its fullest clinical and scientific potential, ensuring reliable diagnostics today and enabling groundbreaking research for the future
